# “I Didn’t Have a Choice”: Experiences, Responses and Perceived Motivations for Reproductive Coercion and Abuse in Australian Women

**DOI:** 10.1177/10778012241292265

**Published:** 2024-10-30

**Authors:** Tiffany Humphreys, Nicola Sheeran

**Affiliations:** 170572 School of Applied Psychology, Griffith University, Brisbane, Queensland, Australia

**Keywords:** reproductive coercion and abuse, reproductive harm, reproductive autonomy, perpetrator motivations

## Abstract

This study explored reproductive coercion and abuse (RCA) experiences of community-based victim/survivors, their responses to RCA, and perceived motivations for RCA. One hundred and one female RCA victim/survivors completed an online questionnaire. Intimate partners, family, friends, cultural/religious leaders, and health professionals were RCA perpetrators. Victim/survivors’ RCA experiences were heterogeneous, incorporating a range of pregnancy-preventing and pregnancy-promoting RCA tactics. Women's response to RCA depended on how it was experienced; victim/survivors were more likely to reassert control when RCA was verbal and not physical. Finally, control was the primary motivator for RCA, followed by contextual factors, drug use, and religion/culture.

Reproductive coercion and abuse (RCA) is a form of violence against women that encompasses pregnancy-promoting and/or pregnancy-preventing behaviors, and is experienced through pregnancy coercion, contraception control, and pregnancy outcome control (Moulton et al., 2021). Pregnancy coercion refers to any form of pressure to become pregnant or prevent pregnancy; contraception control includes any behavior that reduces a person's autonomy over contraception choices and access to contraception; and finally, pregnancy outcome control comprises any behavior that reduces a person's autonomy over pregnancy continuation or termination (Grace & Anderson, 2018; Marie Stopes Australia, 2018). Perpetrators of RCA employ physical, psychological, sexual, economic, and other strategies to reduce women's reproductive autonomy (Moulton et al., 2021).

Victim/survivors of RCA experience a myriad of negative outcomes, such as unplanned pregnancy, sexual and reproductive health disorders, maternal and perinatal ill-health, and poorer mental health (Alexander et al., 2019; Marie Stopes Australia, 2018; Price et al., 2022). Measurement inconsistencies have hindered our understanding of prevalence, however, American studies found prevalence rates between 8% and 30% (Rowlands & Walker, 2019), depending on the research population/setting, sample size, and measures used. Thus far, Australian research has found lower prevalence rates, which appear to also vary based on setting, population, and measurement used, such as 2.3% (Cheng et al., 2021), 5.9% (Price et al., 2022), and 15.4% (Sheeran et al., 2022). For example, Cheng et al. (2021) captured RCA using two screener items from 5497 women attending a community-based nongovernment clinic for a range of reproductive and health concerns in New South Wales. Conversely, Price et al. (2022) sampled from 3,117 women utilizing a nongovernment telephone counseling information service for unplanned pregnancy in Queensland; whereas Sheeran et al. (2022) sampled from 5,107 women accessing support from an Australia-wide and Queensland-based nongovernment organization for past and current pregnancies. For both Sheran et al. and Price et al., RCA was endorsed based on the presence or absence of behaviors noted during the consultation. Although all studies had large samples, Cheng et al. utilized a quantitative method of screening and across the studies, the women accessed services for varying reasons and from different locations. Inconsistencies and poor RCA measurement (discussed further below) likely mean that recorded prevalence rates are underrepresentations.

Despite prevalence inconsistencies, rates of RCA have been shown to increase concurrently with risk factors. International research found RCA risk factors include: being single, prior intimate partner violence (IPV) victimization, low socio-economic status, identifying as an ethnic minority, and being of reproductive age (Grace & Anderson, 2018; Kovar, 2018; Samankasikorn et al., 2019). Similarly, local studies found that women reporting RCA were more likely to be 25–29 years old, previously contacted health services, unemployed, disabled, separated, identified as Aboriginal or Torres Strait Islander, or identifying as culturally and linguistically diverse (Cheng et al., 2021; Price et al., 2022).

Most research into RCA has explored interpersonal RCA, typically by an intimate partner in heterosexual relationships. Interpersonal RCA is “the deliberate action by an individual to interfere with the autonomous reproductive health decision-making of another person” (Marie Stopes Australia, 2018, p. 20). However, an emerging body of research suggests RCA is also perpetrated by family members and individuals outside survivors’ close interpersonal relationships, such as health care workers and cultural/religious leaders (Boyce et al., 2020; Douglas et al., 2021; Gupta et al., 2012; Tarzia et al., 2020). The limited research on RCA perpetration outside of IPV means our understanding of how these perpetrators exert power and control over reproductive decisions is incomplete.

Conversely, there is debate over whether structural experiences of reproductive control should be classified as RCA. Structural RCA can be defined as “social, economic, political and cultural norms, practices and policies that interfere with another person's autonomous decision-making in relation to their reproductive health” (Marie Stopes Australia, 2018, p. 21). For example, structural RCA may include state-sanctioned sterilization, the illegalization of abortion, or abortion prevention policies within religious hospitals (Marie Stopes Australia, 2018). Tarzia and Hegarty (2021) claim that the definition of RCA should not include structural forms, as structural experiences are vastly different from RCA experienced interpersonally. Tarzia and Hegarty (2021) further contend that structural mechanisms contribute to the perpetration and experiences of RCA but are not themselves RCA.

This definitional debate has hindered the exploration of structural RCA. There is little extant literature on how structural RCA is experienced by victim/survivors and the associated outcomes. However, an Australian study has begun to show how interpersonal RCA may intersect with structural forms of RCA. Tarzia et al. (2022) found that Australian women from migrant minority ethnic backgrounds experienced RCA interpersonally by family, cultural/religious leaders, and intimate partners using structural inequalities (e.g., threats to rescind visas) to perpetrate RCA (e.g., coercion to terminate a pregnancy). This study shows further research is urgently needed to extend our knowledge of how structural RCA and intersections between structural and interpersonal RCA may be experienced.

Our understanding of RCA has grown since this specific form of violence was first named as reproductive coercion in 2010, with new forms of RCA (such as pregnancy-preventing tactics) continuing to be articulated (Sheeran et al., 2022). However, our understanding of the range and scope of RCA remains somewhat underdeveloped, as research has largely sampled from niche populations, such as those seeking support from family planning clinics or domestic violence services, and not from broad community-based settings and/or has focussed on specific ages/races (Munoz et al., 2022).

Indeed, the commonly used RCA measurement (i.e., Reproductive Coercion Scale, see Miller et al., 2010) was developed during seminal research from experiences of women presenting to family planning clinics who had previous IPV history, and this measure, or a reduced item version (see Bagwell-Gray et al., 2021; McCauley et al., 2017) remains the dominant RCA measure used in studies around the world. Problematically, this measure does not adequately capture RCA behaviors outside IPV (i.e., family perpetrated, carers, religious and cultural forms of RCA) and does not include items related to pregnancy-preventing RCA behaviors. The lack of pregnancy-preventing items poses a significant problem in RCA measurement, as a recent study of Australian women seeking pregnancy counseling found that around half of the women reporting RCA (15.4%) experienced pregnancy-preventing behaviors (7.5%), and only 1.9% experienced both pregnancy-promoting and pregnancy-preventing concurrently (Sheeran et al., 2022). This highlights that prevalence rates of both current and historical RCA are likely underrepresentative of actual RCA figures.

The few studies (Basile et al., 2019; Munoz et al., 2022; Tarzia et al., 2017) that have sampled from community-based populations have either utilized the RC Scale (Miller et al., 2010) or a two-item screener from the 2010 National Intimate Partner and Sexual Violence Survey (see Black et al., 2011). Unfortunately, this two-item screener does not include behavioral intent. Behavioral intent assists in categorizing behaviors (Tarzia et al., 2020). For example, stealthing (nonconsensual removal of condoms) may occur for a range of reasons, such as increased pleasure or pregnancy promotion. By including perpetrator intent in item wording (e.g., removed condom during sex to get you pregnant) researchers can be certain they are accurately capturing phenomena of interest. Therefore, the abovementioned community-based studies are subject to both specificity (i.e., including individuals whose experiences are not in the context of RCA, such as nonpregnancy-promoting stealthing) and sensitivity (i.e., not capturing all forms of RCA) issues. Consequently, it is unknown if women who are not presenting to service-based clinics experience forms of RCA not captured in current RCA measurement.

Few studies have explored how victim/survivors of RCA respond to experiencing RCA and how, if at all, they attempt to reassert reproductive autonomy. Research exploring RCA responses currently suggests that survivors may use contraception that is harder to detect, such as long-acting reversible contraception methods or emergency contraception (e.g., the morning-after pill), to avoid pregnancy (Moulton et al., 2021). Additionally, American research found once perpetrators were incarcerated, impregnated survivors experiencing pregnancy-promoting RCA sought abortions (Nikolajski et al., 2015). Limited research has shown some survivors of RCA will reach out to others for support, such as family and community services (Moulton et al., 2021). For example, survivors may gain access to forbidden contraception by sourcing medication from friends (Boyce et al., 2020). However, little is known about whether there are other steps survivors of RCA take to exert reproductive control and the contextual factors supporting women to reassert control.

Our understanding of why RCA is perpetrated is in its infancy. Like IPV, RCA is theorized to occur in the context of the need for control and power over the victim (Grace & Anderson, 2018) and instigated by social and cultural drivers (Marie Stopes Australia, 2018). Consistent with this, Moulton et al. (2021) conducted a qualitative synthesis of 13 relevant studies, which found that victim/survivors believed societal (e.g., systemic inequalities) and cultural factors (e.g., male child preference) motivated the perpetration of RCA by family and intimate partners. Although, systemic inequalities as an RCA motivator was dependent on the victim/survivor's race (Moore et al., 2010; Nikolajski et al., 2015). While Nikolajski et al. (2015) found that all women experienced men exerting power and control, only African American victim/survivors recalled that systemic inequalities perpetuated men's motivation for control and power. These women reported that men's impending incarceration and economic instability prompted RCA in a bid to establish relationship and financial security through the victim/survivor via forced pregnancy.

Only one study has explored men's perspective on male RCA perpetration within the context of general childbearing desires in a sample of 23 young Black/African American men. Alexander et al. (2019) found that RCA perpetration was likely influenced by men's childbearing motivations (e.g., the need to extend the immediate family), desires (e.g., romantic connection), and intentions (e.g., abortion resistance), which were driven by social (e.g., economic instability) and cultural factors (e.g., perceived dominance over women). However, as this sample mostly did not endorse perpetrating RCA, it remains unclear whether these findings actually underpin RCA, particularly outside of Black/African American men.

The aim of this study was to expand our understanding of RCA by exploring the experiences of women not presenting to pregnancy, cultural or domestic violence services (i.e., community-based victim/survivors), particularly regarding RCA perpetrated by nonintimate partners and importantly, structural experiences of RCA. Within these experiences, we also aimed to explore how victim/survivors respond to RCA and victim/survivor's perceived motivations for RCA perpetration.

## Method

### Study Design

This study utilized data from a larger Australian research project completed in 2020 focusing on community understanding and experiences of RCA. This data was collected via a cross-sectional online survey with a mixed-method design. A mixed-method design was employed, as it was deemed the most suitable for comparison of RCA experiences against current RCA measurements. Further, a qualitative element, with a phenomenological lens was utilized to extend our understanding of RCA experiences beyond the current quantitative measurement.

### Participants

Participants were Australian community members recruited via convenience methods such as Facebook, flyers, emails to university staff and students, and the psychology first-year subject pool. Exclusion criteria included participants with limited English, aged below 16, who did not identify as female, and those who had not experienced RCA. This resulted in a sample of 101 female participants who endorsed experiencing RCA, with ages ranging between 18 and 62 years (*M *= 29.81 years; *SD *= 9.92). Participant demographics are presented in Table 1. Most participants were born in Australia (84.2%) and lived in the state of Queensland (92.9%), followed by New South Wales (4.1%), Victoria (2%), and Tasmania (1%). Although a representative community sample of participants was sought, compared to the Australian population, women obtained in this sample were younger and less likely to be married (Australian Bureau of Statistics, 2021); consistent with RCA risk factors (Price et al., 2022).

**Table 1.** Demographic Characteristics of Participants.

Variable	Percentage
Ethnicity	
White/Caucasian	82.0
Aboriginal and/or Torres Strait Islander	6.0
Middle Eastern	5.0
Asian	2.0
Pacific Islander	2.0
Mixed	2.0
Hispanic	1.0
Religious or spiritual affiliation	
Not religious	52.0
Religious	48.0
Marital status	
Single	20.8
Casual/noncommitted relationship	3.0
De-facto	24.8
In a relationship but residing separately	22.8
Married	25.7
Divorced/separated	3.0
Employment	
Student	32.7
Employed	63.4
Not employed	4.0
Education	
Year 12 (or equivalent) or below	35.7
Certificate or other qualification	12.9
Advanced diploma/diploma	13.9
Graduate diploma/certificate or bachelor’s degree	25.7
Postgraduate degree	11.9
Annual household income	
$0–$50,000	28.2
$51,000–$100,000	34.1
$101,000–$150,000	20.0
>$150,000	17.6

### Measures

Participants provided age; birth country; residential postcode; ethnicity; annual household income; educational attainment; and religious, employment, and relationship status.

#### Presence of RCA

Two dichotomous items were provided to participants throughout the questionnaire to identify RCA. Participants were asked to identify RCA firstly via the following item: “After reading the definition of RC, have you have ever experienced this first-hand (directly happened to you) yes/no.” Positive response to this item led to an open-ended response box with the following instructions “In the last question you stated that you had experienced RC in some form. In the box below can you please tell us in as much detail as you feel comfortable with, your experiences with reproductive coercion.” Participants were again provided with the definition later in the questionnaire and asked “Considering the above definition, do you think you have ever experienced RC? Yes/No.”

Participants also completed a 13-item measure based on the RC Scale developed by Miller et al. (2010)—see Table 2 for item wording. The original scale included six items measuring pregnancy coercion and five items measuring birth control sabotage. The variables are binary (yes/no) and endorsement of any of the 11 items signifies the occurrence of RCA. Two additional items were included in the measure to incorporate contemporary research on RCA experiences. Additional items included “refused to pay for birth control/condoms because they wanted/desired pregnancy” (Sutherland et al., 2015) and “made you terminate a pregnancy that you wanted to continue with.” The original scale has shown consistent moderate internal reliability through multiple published studies, ranging from .66 to .76 (Grace & Anderson, 2018). With the adaptations, this study found good internal reliability (*α *= .84).

**Table 2.** Endorsement Frequencies for RCA Behaviors.

Item	Yes %
Pregnancy-promoting RCA	
Told you not to use any birth control (like the pill, shot, ring, etc.)?	36.5
Tried to force or pressure you to become pregnant?	28.1
Made you have sex without a condom so you would get pregnant?	21.8
Taken off the condom while you were having sex so that you would get pregnant?	15.6
Refused to pay for birth control because they wanted/desired pregnancy?	13.5
Hurt you physically because you did not agree to get pregnant?	11.5
Told you they would have a baby with someone else if you didn't get pregnant?	10.4
Taken your birth control (like pills) away from you or kept you from going to the clinic to get birth control so that you would get pregnant?	10.4
Said they would leave you if you did not get pregnant?	9.4
Made you hide birth control because you were afraid they would get upset with you for using it?	9.4
Broken a condom on purpose while you were having sex so you would get pregnant?	5.3
Put holes in the condom so you would get pregnant?	3.1
Pregnancy-preventing RCA	
Made you terminate a pregnancy that you wanted to continue with	20.8

*Note.* RCA: reproductive coercion and abuse.

#### Perpetrator

After completing the RC Scale participants were then asked “You stated that you may have experienced controlling behaviour. Please indicate from the following who behaved this way towards you: a. intimate partner; b. family member; c. important religious figure; d. important cultural figure; e. important community figure; f. other.”

#### Open-ended questions

To capture RCA experiences, participants were asked several open-ended questions about their RCA experience. For example, one open-ended question assessed experiences of RCA not captured in the scale above by asking, “Is there any other behavior that you have experienced that you would think comes under reproductive coercion not listed above?,” where a dichotomous yes/no answer was provided along with a prompt where participants were asked to describe their experience in an open-ended response box.

**Motivations underpinning RCA**. To understand what victim/survivors believe to be the reason behind their experience, participants were asked: “We would like you to think back on the behaviors that you experienced and what you perceived the underlying motivations for this behavior to be. In the box below, please describe why you think this person behaved this way towards you, and what their motivation/goal was. This does not suggest in any way that you caused this behavior—we are attempting to understand how people make sense of the way other people act.”

**Actions to retain/regain control.** To understand how victim/survivors respond to RCA, participants were asked: “We are interested in how people respond to experiencing reproductive coercion. Above you stated that you experienced behaviors consistent with reproductive coercion. In the box below, please describe, if relevant, any way you responded to the behavior. For example, complied with the request, took contraceptives in secret etc.”

#### Procedure

Ethical approval was granted by Griffith University Human Research Ethics

Committee (Ref No: 2020/067). The online survey hosted on LimeSurvey took 20–30 min to complete. Participants were offered course credit if they were first-year psychology students or the chance to win one of six $50 gift cards. Prior to commencing the online survey, participants were provided with a summary of the study to facilitate informed consent. This information sheet included an overview of the study purpose, estimated completion time, and a statement of risk. It was highlighted to participants that there was minimal participant risk, however, some participants may experience discomfort surrounding the nature of the questions asked. It was made clear to participants that their participation was voluntary and that they may withdraw at any time prior to submission. No deception was used throughout the survey and participants received clear instructions/information regarding each topic measured. Throughout the survey participants were also reminded that they could close the survey down at any time. Participant distress rating was measured via a Likert-type scale at multiple time points during the survey with recommendations to cease the survey if high distress ratings were endorsed. Relevant service numbers were provided at the end of the study to support participant wellbeing.

### Analytic Strategy

Quantitative data was analyzed using IBM SPSS 29. Descriptive and frequency analysis was utilized to identify participant demographics and endorsement frequencies within the 13-item RCA measure. From the participant pool of 101 victim/survivors, 99 provided qualitative accounts of their RCA experience, response, and perceived perpetrator motivation through the open-ended survey questions. Responses varied in length and quality and some women answered all questions, whereas others responded to just one or two. Participants open-ended responses were entered into NVivo Version 12 and analyzed using inductive descriptive content analysis (Braun & Clarke, 2006; Vears & Gillam, 2022). The first author commenced by familiarizing themselves with the participants’ qualitative responses. Narrow content categories were then developed (e.g., types of RCA behaviors, motivations, and responses). Following an iterative approach, content codes were grouped where appropriate to create overarching content categories. Subsequently, manifest content was reviewed, and prominent latent themes were identified (Graneheim et al., 2017). The final themes were refined by the first and second authors. It is important to note the subjectivity in analyzing qualitative data; while the authors aimed to be as objective as possible, both authors are white clinical psychologists and researchers.

## Findings

The findings are presented in three sections. The first explores how victim/survivors experienced RCA, which includes a descriptive analysis of the RC Scale item endorsement, perpetrators, and RCA behaviors categorized by perpetrator. The second section includes findings related to how victim/survivors responded to RCA and consists of four themes: retaining reproductive autonomy, confrontation and passive resistance, women reexerting reproductive control over men, and compliance with RCA demands. The final section provides findings on victim/survivors’ perceived motivations for RCA perpetration and consists of four themes: control, contextual factors, drug use, and religion/culture.

### What Did Victim/Survivor's Experience?

From the total pool of participants, 73 endorsed at least one item on the RC Scale. Endorsement rates for the RC Scale are shown in Table 2 and ranked from most to least endorsed. Participants could endorse more than one item, the mean number of item endorsements was 1.94 (*SD *= 2.42), with a minimum of 0 and maximum of 11 item endorsements. A total of 52.5% of the sample reported experiencing at least one form of pregnancy coercion on the RC Scale, 27.7% endorsed at least one type of contraception sabotage, and 20.8% reported pregnancy-preventing RCA.

**Figure 1.**
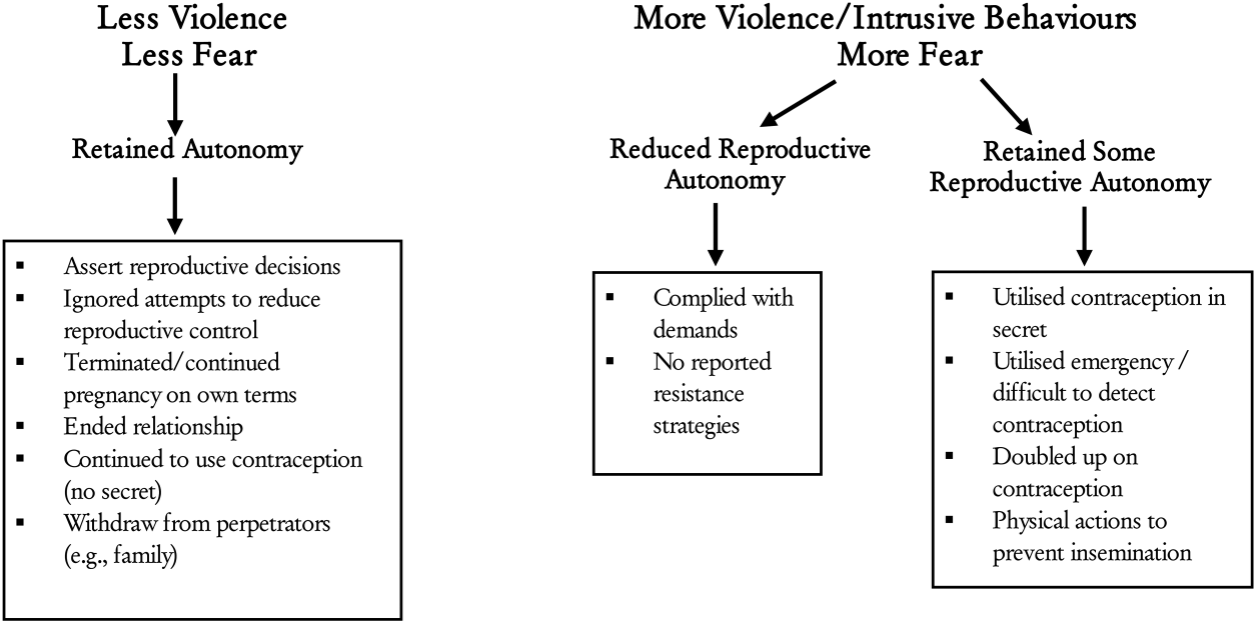
Impact of violence continuum on survivor/victim response.

Perpetrators were identified by 91 participants. Intimate partners were most common (75), followed by family members (9), important community figures (2), important cultural figure (1), and important religious figure (1). Three participants reported “other” perpetrators: including concurrent partner and family RCA, society, and the government.

#### Additional RCA behaviors/tactics by intimate partners

In their qualitative responses, participants reported behaviors by intimate partners that were not captured by the amended RC Scale (see Table 3); this was particularly notable for pregnancy-preventing forms of RCA. Further, there was a temporal and ever-changing nature to participant's accounts of RCA behavior. When experiencing pregnancy-promoting RCA by intimate partners, participants recounted that pregnancy pressure would lead to pressure to not use contraception. Once pregnant, they experienced pregnancy outcome control through coercion to prevent pregnancy termination. Conversely, for other participants, both long-acting methods and the contraceptive pill were forced upon them in a bid to prevent pregnancy. Once pregnant, their partners tried to ensure pregnancy termination through coercive methods such as psychological abuse or threats of harm.

**Table 3.** Examples of Additional Intimate Partner RCA Behaviors.

Specific RCA behavior	Exemplar quotes
Pregnancy-promoting RCA	
Pressure/control of pregnancy-promoting aids	The pressure was to continue with IVF treatment and I wanted to stop. (P604)
Contraception control: hiding contraception	My ex pressured me into kids and would hide my pill to stop me from taking it. (P825)
Pregnancy outcome control: abortion prevention	My past DV relationship of 2 years included an abortion that he insisted on and we agreed. He was controlling and aggressive so I was worried to keep it. Then would tell me he wanted it and then he told his family which I said would make it harder before the procedure, he would say things like you have my baby inside of you and you took my child away from me and I wanted to have kids with you. (P812)
Pregnancy-preventing RCA	
Threats to prevent pregnancy	Was told that if I ever fell pregnant that I would have to get rid of it and they would leave me. (P286)
Forced contraception	My ex was in charge of my birth control, he forced myself to have the “bar” put in when I was 15, (he was older then myself), he drove me to the appointment, pretended to be my care giver and paid for the appointment. (P254)
Physical violence to terminate pregnancy	I was kicked in the belly while I was pregnant. (P382)

*Note.* RCA: reproductive coercion and abuse.

#### Family RCA perpetration

Family RCA was perpetrated by parents and in-laws. Several participants reported experiencing RCA from partners and family concurrently, such as Participant 781 “I was forced into terminating a pregnancy when i [sic] was younger by my partner at the time and my family.” RCA perpetrated by family included both pregnancy-promoting and pregnancy-preventing RCA. See Table 4 for examples of family perpetrated RCA.

**Table 4.** Examples of Family Perpetrated RCA Behaviors.

Specific RCA behavior	Exemplar quotes
Pregnancy-promoting RCA	
Pressure to fall pregnant	Prolonged and excessive pressure from parents in law to become pregnant to make their family complete. Mother-in-law bought herbal supplements and expected (actually, she demanded, had tantrums when he didn't want to talk to her about why he wasn't taking them) my husband to consume them to bring a grandchild into her life because all their friends had grandchildren to talk about and visit. (P673)
	My father is very controlling and abusive. He has told me many times to get pregnant at age 25 and it MUST be a boy or else I'm in trouble. I just try to ignore my father's demands because it's my body and I should get to choose when I'm ready for a child not when he wants a grandson. My father wants a grandson because my family has very old traditions of women stay at home and men work. He has always hated me because I don't comply with my families beliefs. If I am to provide him with a grandson by the age of 27 my son will be allowed to be a part of his family as I'm considered an outcast. (P441)
Contraception prevention	Living in a very religious household, contraception is not allowed. Even for period relief (as i suffer from endo and have extreme periods), the pill or condoms are always thrown out if found in your room. (P477)
Pregnancy-preventing RCA	
Pressure to terminate pregnancy	Falling pregnant while in year 12, my mother made it clear she was ashamed of me and made it difficult and uncomfortable to discuss practicalities and options or to work out what outside services I may access to help make a decision. She made an appointment for termination for me and I went along with it, I was scared and confused and didn't know what to do. I thought to regain my place in her eyes that it was I needed to do. (P463)
Forced contraception	In a way, my father decided over my body when I was 14 years old, he forced me to insert the Implanon even though I did not understand what it was and how it affected my mental health and body, plus it was a big commitment and my body hasn't been the same since then, I do not have periods and probably it will take me a long time to have a baby when I decide to have one. (P371)

*Note.* RCA: reproductive coercion and abuse.

#### Structural RCA

Participants reported feeling coerced or controlled by religious and government institutions regarding reproductive decisions. Some participants were able to maintain control of their reproductive decisions, and others were not. For Participant 227, religion and government institutions combined to control her reproductive choices:

Governments denying or criminalizing abortion, and communities not allowing them to be accessible. I took contraceptives against religious advice … I also imported contraceptives when living in a country that banned them (Philippines).

Several participants described how their religion covertly and/or overtly attempted to control their reproductive decisions. Some participants spoke of religious teachings and culture pressuring against using contraceptives, away from abortion, or toward childbearing:

Verbal RC by family, friends … friends of friends who I barely know, religious figureheads (this is general advice to the Muslim women, I was not targeted personally but still felt upset by it), my husband would love more children but is being patient with me regarding this. (P806)

Participants’ experiences also highlighted the fine line between doctors’ advice and doctor coercion regarding reproductive decisions. Three participants experienced medical professionals withholding information, leading to reduced reproductive autonomy and negative outcomes. All three women presented to their doctor to explore pregnancy-prevention strategies, and all three experiences reflected feeling dismissed and unsupported. For example, Participant 701 reported feeling coerced into a specific reproductive decision “I found out about IUDs from the internet, and my doctor at the time (male) tried to dissuade me from it because I was in my ‘prime baby years’.” For Participant 717, denial of options led to psychological stress due to fear of becoming pregnant:

I do not want children. … I went to see doctors about steralisation [sic] and they said I was too young to make decisions that I would or would not want children. This occurred until my mid 30 s. I was advised and tried a number of birth control methods such as implanon and an IUD and the pill which all had negative effects on my health …. I do not share that I feel relief about my partner's impotence however I feel that our relationship is better because of it and hope that this relationship will last.

### Response to RCA

Participant's experiences of RCA fell upon a continuum of violence and fear which influenced whether victim/survivors retained reproductive autonomy or not. Despite initially complying with demands, often for safety, some victim/survivor's employed hidden resistance strategies to maintain some reproductive control. See Figure 1 for a visual representation of the impact of the violence continuum on victim/survivor responses.

#### Theme 1: Retaining reproductive autonomy

Some experiences appeared to be characterized by a lack of fear and perpetrator control, resulting in retained reproductive autonomy. For example, after indicating their partner had said he would leave her if she did not fall pregnant on the RC Scale, Participant 800′s qualitative response implied this occurred without the presence of fear and control. This participant indicated they did not classify the behavior as “abusive,” raising questions as to where the threshold for “pressure” becomes experienced as “abuse”:

We continued our relationship after a conversation which stated I would not have a child, they then said they still wanted one and in the future they could potentially leave me for someone that did want one. I agreed to this. if you want a child, see someone who wants one. we were both young at the time though … I wouldn't really concider [sic] it RC …. but it does appear that way with your definition.

#### Theme 2: Confrontation and passive resistance

Participants reported confronting the perpetrator and resisting reproductive control when the RCA was less violent, such as verbal pregnancy pressure.

I highlighted the hypocrisy of his attitude, which was underpinned by religion and gendered beliefs. This hypocrisy led to him believing that it was ok to be sexually active, but not ok for the women he was having sex with to protect themselves against pregnancy. I ignored his disapproval, and continued to use contraception. (P743)

Rather than confronting the perpetrator directly, other women ignored and resisted contraceptive control, “I was told not to take the morning after pill. Which I processed [sic] to do so” (P703) and pregnancy pressure “I often laughed it off, or pretended to like things I did not to reduce conflict … luckily he eventually stopped trying to convince me when he suddenly decided he didn’t want a child” (P593).

Other women continued the pregnancy despite pressure for termination “I was begged to have an abortion but stayed my ground and had the baby” (P844), or terminated pregnancies on their terms “This decision was hard … This to me, was my only option to protect myself and the baby” (P760). Family perpetration led to passive resistance and withdrawal “I withdrew from family events and had high levels of ongoing stress” (P673).

#### Theme 3: Women reexerting reproductive control over men

A portion of participants complied with perpetrator demands, however, sought to secretly regain control where possible. Participants experiencing contraception control often took contraceptives in secret, utilizing concealable oral and long-acting contraception. For example, Participant 664 recounted she “Had an implanon put in secretly but complied with request to not use condoms out of fear for being the physically punished.” When Participant 825′s partner hid her pill and pressured her into having children she doubled up on contraceptive methods “My ex used to hide my pills so I couldn't take them. To ensure I didn’t get pregnant I got a contraceptive bar in my arm because I knew there was no way he could take it from me.” One woman sought alternative fertility prevention options “In other cases I organized contraceptives without my partner knowing. I also used herbal medicines that are supposed to reduce fertility and the ‘morning after pill’ in secrete [sic]” (P717).

For participants who experienced pregnancy-promoting behaviors emergency contraception was utilized in an effort to prevent forced pregnancies, such as Participant 616 “I complied at the time as I didn't have a choice. Afterwards I went to the doctor for the morning after pill.” Another woman enlisted more physical tactics to prevent possible impregnation. Participant 812 experienced pregnancy-promoting RCA through sexual violence and was later prevented from terminating her pregnancy. To reexert control, she attempted to prevent the perpetrator from ejaculating inside of her through moving her body prior to ejaculation:

I would pretend to be asleep a lot but that usually wouldn't stop him, I would just have to make sure I was positioned in a way that I could push him off me if I could feel that he wasn't pulling out.

#### Theme 4: Compliance with RCA demands

Despite the examples above, participants most commonly stated they did what the perpetrator demanded of them as they had no other option, such as in Participant 29′s experience “I wanted to keep my pregnancy but he didn’t want to have a child at the time. I regret having the termination and I felt as if I didn’t have a choice.” This was particularly prevalent in more violent forms of RCA, where there was cooccurring perpetration of IPV. These participants feared retribution or violence for resisting and some participants reported that compliance was the safest option. For example: “Had no choice but to take the punches to my stomach area for him to try kill the baby. I would either be forced down or drugged up by him” (P45). Participants also highlighted the significant effort required to challenge RCA. For example, Participant 760 recounted:

I learned very quickly that if i [sic] tried to debate the point, express my opinion or concerns that it would not end well for me. The easiest and safest thing for me to do was to comply with the request. If i [sic] were to fight back, he would usually get what he wanted by force.

### Perceived Motivations for RCA Perpetration

The most common perceived motivation for RCA was control. However, three further themes were also identified: contextual factors, drug use, and religion/culture (see Table 5).

**Table 5.** Examples of Perceived Motivations for RCA Perpetration.

Theme	Exemplar quotes
Control: partner	
Relationship/family promotion	They didn't want me to leave the relationship so they thought that getting me pregnant would give them a permanent connection with me. (P117)
Self-centered	They did not want to ever have children, but they were to selfish/scared to tell me, so they continued to make me terminate pregnancies (2). (P671)
Contextual factors: partner	
Losing children	My ex's ex moved overseas and took his kid with her. I believe my ex was trying to coerce my into getting pregnant so he could ‘replace’ his child/cope with feelings of abandonment. (P825)
Trauma	Because he was a violent asshole. Had a very violent childhood, beaten by father, made to go to the army and kill people at the age of 17 (on the day his father died), his culture and religion (Afrikaans—fundamentalist Christianity) emphasizes male dominance and patriarchal power. (P721)
Mental health	Partner was going through depression and was worried if I went through with the pregnancy it would depression would turn suicidal. (P755)
Drug use	He hated himself. he was a miserable sad drug addict. He did not want to pay for a baby. (P780)
Culture/religion	
Religion: partner	He did not believe women should use contraception on religious grounds mainly. (P743)
Culture: partner	Expectations about relationship—he is from Pakistan where having kids is a crucial part of marriage and heritage. Not wanting/having kids is not something common. (P613)
Religion: family	Growing up in the Catholic faith the unspoken understanding in my family was that if I was to fall pregnant no matter what I wanted, the option of termination was not a consideration. (P690)

*Note.* RCA: reproductive coercion and abuse.

#### Theme 1: Control

Overarchingly, participants who experienced intimate partner RCA believed the motivation for the abuse was gaining control over them. However, underlying control, two clear themes emerged between motivation and RCA type. Participants who experienced pregnancy-promoting RCA were more likely to report that their perpetrator wanted children or believed pregnancy would ensure relationship continuation. However, participants who experienced pregnancy-preventing RCA assumed the desired control was due to the perpetrator being self-centered (e.g., forcing contraceptives as they refused to wear condoms), or not wanting to give up their lifestyle/not ready for children (e.g., forced termination).

#### Theme 2: Contextual factors

Some participants also reflected on the contextual factors underlying their partners’ RCA. Participants recounted that historical trauma, the impact of previous relationships, and losing contact with children due to a former partner likely fostered pregnancy-promoting RCA perpetration. In particular, the men with children to prior partners enacted pregnancy-promoting RCA through pregnancy coercion and hiding contraception to lessen the psychological impact of previous relationships ending. One woman recounted that her partner began pressuring pregnancy almost immediately upon commencement of the relationship “He already had two children to a previous marriage and I think he just wanted to show her that he could move on and have babies with another young, successful woman” (P791).

Further, the perpetrator’s poor mental health was described as a contributing factor in participants’ RCA experiences. Two participants thought their partners pressured pregnancy termination due to the perceived negative impact that continuing with the pregnancy would have on their (the perpetrator’s) mental health. Conversely, one woman felt that her partner’s significant low self-esteem contributed to his need to feel unconditionally loved by her and a child, which led to pregnancy-promoting RCA via coercion to become pregnant, contraception control, and psychological abuse to prevent pregnancy termination. Finally, for one woman, her partner’s history of trauma, culture, and religion all combined to increase RCA perpetration (see Participant 721 in Table 5).

#### Theme 3: Drug use

Several participants hypothesized that their intimate partners’ illicit drug use led to RCA perpetration. Most participants who indicated illicit drug use as a motivator experienced pregnancy-preventing RCA, particularly forced abortion. These women experienced a range of behaviors to ensure termination, including psychological abuse, forced maternal illicit drug usage, and physical abuse. Most of these women reported that their partners did not want a child to impede their (the perpetrator’s) ongoing drug use and lifestyle. However, one woman recounted that while her partner’s drug use contributed to his behavior, the forced termination was so the perpetrator could have ongoing control over her, rather than focusing on his ongoing lifestyle drug use as noted by the previous women. For example:

When my ex and his mother-in-law forced me to go for an abortion even though it wasn’t what I wanted. It was at a threat of physical harm if I didn’t comply. They drove me in and then came into the appointment so I couldn’t even ask for help there. It was an abusive relationship. He was mentally unstable because of drug use and it was always about controlling me. A child would have taken me away from him and he could never tolerate that” (P625).

Only one woman recounted that she experienced pregnancy pressure while her partner was consuming drugs, noting the perpetrator only wanted pregnancy so his mother would fund his drug habit.

#### Theme 4: Culture and religion

Culture and religion were also cited as motivators, particularly for participants who experienced pregnancy-promoting RCA or RCA by family members. These women often noted the interplay between male gender dominance within the perpetrators’ culture/religion and controlling behavior. Additionally, the line between RCA behaviors (i.e., contraception control) and religious adherence within families was blurry in some participants’ experiences.

## Discussion

The aims of this study were threefold: to explore how RCA was experienced by women in the general community, how they responded, and, what they perceived motivated the RCA. Our findings suggest that intimate partners, family, friends, cultural/religious leaders and organizations, and health professionals can be perpetrators, and that experiences of RCA are heterogeneous, incorporating both pregnancy-preventing and pregnancy-promoting RCA. Forced contraception, which is poorly articulated in research and typically not included in RCA measures, was prominent. Our findings suggest women, particularly young women, may experience forced contraception by family and partners. Further, we found that victim/survivors were more likely to reassert control when RCA was verbal and not physical and the type of RCA influenced how victim/survivors reasserted control, with contraception and morning-after pills commonly used when faced with pregnancy-promoting RCA. Control was seen as the most common motivation for the perpetration of RCA, followed by contextual factors, drug use, and culture/religion.

### RCA Experiences of Community-Based Women

The findings of this study suggest RCA is most commonly perpetrated by intimate partners, however, parents/in-laws, doctors, and government and religious institutions also exert reproductive control. This supports earlier work highlighting that RCA is broader than IPV, while also extending our current RCA knowledge by providing clear evidence of the impact of structural RCA through women's lived experiences (Senderowicz, 2019; Tarzia et al., 2020). Women experienced RCA from multiple perpetrators concurrently, which has also emerged in recent studies with migrant and refugee women (Tarzia et al., 2022). However, far less is known about the dynamics that underpin multiperpetrator RCA, nor how it intersects with other forms of violence (i.e., family). Health professionals are also emerging as common perpetrators or parties who are at least complicit in RCA (Senderowicz, 2019). More research is needed to understand how and why these professionals exert control.

Consistent with other community-based studies, pregnancy coercion, and contraception control/sabotage were the most common forms of RCA experienced by those in our community sample (Basile et al., 2019; Munoz et al., 2022; Tarzia et al., 2017). This study joins an emerging body of research that suggests pregnancy-preventing RCA, in the form of forced termination, may be as prevalent as pregnancy-promoting RCA, such as condom removal to cause pregnancy. Accordingly, Sheeran et al., (2022) found pregnancy-preventing RCA was marginally higher than pregnancy-promoting RCA (9.4% vs 7.9%).

However, due to limited RCA research and the RCA measure used, only one quantitative item was included in this study measuring pregnancy-preventing RCA, in the form of forced termination. Contemporaneous research has since highlighted additional categories of pregnancy-preventing RCA, including forced contraception/sterilization, covert administration of abortifacient agents, and physical violence to induce miscarriage (Rowlands et al., 2022). It is unclear whether the endorsement rates for pregnancy-preventing RCA against pregnancy-promoting RCA may have changed if these additional categories were quantitatively measured, in addition to being captured qualitatively.

As anticipated, we identified additional behaviors in our qualitative data that constitute RCA, particularly for pregnancy-preventing RCA. This included threats to prevent pregnancy before pregnancy occurs, forced contraception usage, and forced pregnancy terminations. Forced contraception usage and pregnancy terminations have been presented in international (Moulton et al., 2021) and Australian research (Suha et al., 2022; Tarzia et al., 2020), however, to the authors’ knowledge, threats to prevent pregnancy have yet to be documented in qualitative RCA research. Together, these findings highlight the importance of assessing both forms of RCA and for a more comprehensive RCA measure than what currently exists.

### Victim/Survivor Response to RCA

While our understanding of women's response to RCA remains in its infancy, our findings are consistent with extant research suggesting RCA victim/survivors employ tactics to regain reproductive autonomy (Moulton et al., 2021). However, we also found the degree of violence and subsequent fear induced appears to influence women's responses. Our findings suggest women who experienced verbal RCA were more likely to report reduced fear and consequentially, increased reproductive assertiveness. These women retained their reproductive autonomy by employing a range of resistance strategies (e.g., verbal assertiveness, ignoring demands, leaving a relationship, continuing/ending a pregnancy, etc.) despite attempted verbal pregnancy pressure, contraceptive control, and/or pregnancy outcome control. On the other end of the continuum, victim/survivors who experienced high levels of violence and more intrusive control tactics (e.g., forced contraception) reported increased fear and decreased reproductive autonomy and assertiveness. Thus, the women's experiences suggest that the level of violence influences RCA victim/survivors’ responses.

Despite complying with perpetrator's demands, often to ensure safety, we found that those experiencing pregnancy-promoting RCA were sometimes able to access concealable contraception (i.e., emergency/long-acting) to prevent unintended pregnancy. This is consistent with earlier studies identifying resistance tactics against pregnancy-promoting RCA (see Moulton et al., 2021). However, it remains unclear whether women experiencing pregnancy-preventing RCA (i.e., forced contraception/termination) can assert their reproductive control. Women in this study who were forced to take contraceptives (particularly intrusive forms such as the Implanon or injection) all indicated they “complied” with the request without any examples of how they were able to reassert control. Similarly, few women acknowledged asserting control when faced with forced abortions; those who did either separated from the perpetrator or continued the pregnancy. Thus, more research is needed to understand whether there are safe strategies women can employ when facing pregnancy-preventing RCA that do not impact their safety. It may also be important for health professionals to be aware of pregnancy-preventing RCA and its forms, so they can ensure the patient is freely consenting to the procedure.

### Perceived Motivations Underlying Perpetrator RCA

This study extended the limited research on RCA motivations by purposely exploring perceived motivations in a sample of community-based victim/survivors. Supporting Marie Stopes Australia (2018) and Moulton et al. (2021), both social and cultural drivers were found to underly RCA in our study. Control was most referenced as the primary motivator for intimate partner-perpetrated RCA; however, extending our understanding, this paper revealed a possible connection between the type of intimate partner RCA (e.g., pregnancy-promoting vs pregnancy-preventing) and underlying motivation/driver. Control was established by intimate partners for continuation of relationships or to begin a family (pregnancy-promoting), or conversely, to perpetuate their lifestyle or for pleasure (pregnancy-preventing).

Social drivers appeared to motivate RCA outside of intimate partner-perpetrated RCA. Parents enacted pregnancy-preventing RCA based on social expectations of their children (e.g., forced abortion/forced contraception). Similarly, medical professionals’ beliefs about women's reproductive decisions led to the removal of reproductive choices for women.

Culturally, the line between adherence to religion and RCA behaviors was blurry, particularly for pregnancy-promoting RCA. Women reflected that expectations around childbearing steeped in religious and cultural expectations led to the removal of their reproductive rights—by family, religious leaders, friends, and intimate partners. This finding supports Australian studies by Suha et al. (2022) and Tarzia et al. (2020), which also found that cultural and religious views were significantly related to the perpetration of RCA for women.

Contextual factors such as trauma, reduced contact with children, poor mental health, and drug use were hypothesized as motivations for RCA, which has not previously emerged in RCA literature. However, research has shown perpetrators of IPV are more likely to have mental health disorders (Yu et al., 2019). Similarly, extant IPV literature has consistently shown substance abuse and historical trauma (particularly childhood abuse/violence) as strong predictors of male IPV perpetration (Clare et al., 2021). From our knowledge, this is the first study to highlight the underlying psychological factors driving perpetrators of RCA. More research is needed to understand how mental health and trauma contribute to a person's desire to control another's reproductive choices.

Finally, our findings partially support previous research as establishing ongoing ties to women was a perceived motivator, however, this was not necessarily due to incarceration as found by Nikolajski et al. (2015). Although, this is likely due to sampling differences, as it is unlikely that many of our sample had partners who had incarceration pending.

### Implications for Practice and Theory

Similarly to Suha et al. (2022) and Tarzia et al. (2020), we found what appears to be an intersection between interpersonal RCA and structural RCA. Women experienced RCA interpersonally, perpetrated by family, important community leaders, and medical professionals acting under structural entities such as religion and government policy. Women's experiences highlighted how religious leaders can influence partners and reproductive outcomes—against women's wishes. Similarly, doctors’ unwilling to prescribe medications, offer comprehensive reproductive advice, or complete surgical procedures led to the loss of reproductive autonomy for women. Emerging evidence suggests intersecting interpersonal and structural RCA can have significant negative impacts on women (Senderowicz, 2019); yet much of the research to date has focused on intimate partner RCA and our understanding of structural RCA is woefully limited.

As expected, women's qualitative responses indicated an increased taxonomy of RCA behaviors than included on preexisting RCA measures (i.e., RC Scale; Miller et al., 2010), particularly for pregnancy-preventing RCA. This study has shown that to validly measure RCA in a community sample, a quantitative measure must include both pregnancy-promoting and pregnancy-preventing items, which apply to a range of perpetrators, across a range of settings. Similarly, qualitative experiences in this study highlighted the RC Scale (Miller et al., 2010) does not accurately capture the severity of RCA behaviors. As the RC Scale (Miller et al., 2010) items are responded to in a presence or absence binary manner (i.e., yes/no) women in this study were forced to endorse items, even when the RCA was not perceived as abusive. Currently, there is no available scale/assessment that validly measures RCA. Inconsistency and reduced validity in measurement are leading to inconsistent prevalence data, replication difficulties, and possibly erroneous conclusions; therefore, an updated quantitative measure of RCA must be developed.

Our findings support the need for easy access to emergency, oral and long-acting contraception, and pregnancy terminations to assist women to reassert reproductive control. However, this study joins several others (see Alhusen et al., 2020; Sheeran et al., 2022) in highlighting the pressing need for helping professionals (medical and nonmedical) working in sexual and reproductive health, including contraceptive and abortion care, to be aware of RCA indicators and be competent at sensitively exploring RCA with women. Stronger community awareness is needed to support early identification in both family/friend systems.

### Limitations and Future Directions

Despite the significant strengths of this paper, it was not without limitations. While we sought a representative community sample of women, the final sample differed in some key aspects. For example, participants were required to speak English, which likely means the study was underrepresentative of culturally and linguistically diverse participants. This is particularly unfortunate given the reported experiences of family and religion/culture RCA in this study. Future studies would benefit from greater diversity to allow exploration on the influence of religion/culture, family perpetration, and intersections with family violence.

This study specifically explored RCA experiences of women/individuals assigned female at birth. This meant that motivations for RCA were hypotheses and interpretations of men's behavior. At times this was difficult for survivor/victims due to experiencing multiple concurrent forms of abusive behavior within the context of IPV. To implement interventions preventing RCA and reduce ambiguity, future research needs to explore perpetrator motivations from perpetrators of RCA.

## Conclusion

Women in the community are experiencing RCA by a range of perpetrators, employing a range of behaviors not captured on current measures of RCA. Women experiencing pregnancy-promoting RCA sometimes employ strategies to reassert control, such as using hard-to-detect contraception. However, those experiencing pregnancy-preventing RCA, such as forced contraception, appear to have fewer options. Health professionals, particularly those working in sexual and reproductive health, need to be able to sensitively screen for RCA, to avoid creating unnecessary barriers for victim/survivors reasserting reproductive autonomy, along with providing appropriate referral and support options. To assist professionals in identifying those most at risk of RCA, an updated and broader measure of RCA is needed.
